# Variation in transitional care implementation patterns for older adults with stroke in Japan: A retrospective observational study

**DOI:** 10.1097/MD.0000000000048161

**Published:** 2026-03-27

**Authors:** Naoki Takashi, Misaki Fujisawa, Tomoko Ohura, Joji Onishi, Shosuke Ohtera

**Affiliations:** a Department of Health Economics, Center for Gerontology and Social Science, Research Institute, National Center for Geriatrics and Gerontology, Obu, Aichi, Japan; b Evidence-based Long-term Care Team, Center for Gerontology and Social Science, Research Institute, National Center for Geriatrics and Gerontology, Obu, Aichi, Japan.

**Keywords:** conditional inference tree, ischemic stroke, latent class analysis, transitional care

## Abstract

Transitional care is a critical component of healthcare quality and patient outcomes. This retrospective observational study aimed to descriptively identify transitional care service implementation patterns for patients with ischemic stroke in Japanese acute care hospitals and examine their associations with hospital-level characteristics using the Japan Medical Data Center hospital-based claims database. A total of 14,129 patients aged ≥40 years admitted for ischemic stroke and discharged from Japanese acute care hospitals between April 2022 and August 2023. We examined 10 transitional care services from billing claims and grouped them into 4 domains. These 4 binary indicators were used in a Latent Class Analysis to identify unobserved implementation patterns. Conditional inference tree analysis, incorporating hospital- and patient-level variables, was applied to assess characteristics associated with each pattern. Sensitivity analyses using fractional logit regression were conducted to address uncertainty in pattern assignment. Three distinct transitional care service implementation patterns were identified. Two patterns emphasized patient/family education and information transfer, while the third involved resource-intensive services, including discharge planning and structured coordination with post-discharge providers. These patterns were significantly associated with the number of beds and patient age (all *P* < .01). Sensitivity analyses confirmed the number of beds as a consistent, significant factor across patterns. Transitional care service implementation patterns varied across hospitals, likely reflecting structural characteristic differences, particularly hospital size. Larger hospitals tended to implement more comprehensive combinations of transitional care services. These findings highlight how structural characteristics may contribute to variation in transitional care service delivery.

## 1. Introduction

Transitional care is increasingly recognized as a critical component of healthcare quality and patient outcomes, particularly during the vulnerable period of hospital-community transition.^[1]^ However, fragmentation at this stage has been associated with adverse events, preventable readmissions, and greater healthcare utilization.^[2,3]^ International evidence indicates that transitional care interventions, such as discharge planning, patient education, and care coordination, can improve outcomes and reduce costs.^[4-7]^

Hospitals in both the United States and Japan implement diverse transitional care strategies. The structure and delivery of these services in the United States vary considerably across institutions, influenced by resource availability, organizational capacity, and regional healthcare system characteristics.^[8-10]^ In Japan, transitional care is supported through add-on payments under the universal health insurance system, which provides reimbursement incentives for discharge planning, interprofessional collaboration, and information exchange with post-acute providers. Japanese policymakers view transitional care as a core element of integrated care linking medical and long-term services.^[11]^ However, evidence on how these services are structured and delivered in real-world Japanese hospitals remains limited.

As the population ages and some regions undergo depopulation, healthcare demand is projected to decline in certain areas.^[12]^ In response, Japan’s Community Healthcare Vision – a nationwide regional reform initiative developed in 2014 – reached a key milestone in 2025, emphasizing clearer role differentiation among hospitals according to local needs and promoting coordination across care settings.^[13]^

Within this policy context, understanding how transitional care services are delivered and how service combinations vary across hospitals is essential. These insights can help clarify hospital roles within the regional continuum, guide resource allocation, and address disparities in access to transitional care.

This study focused on patients with ischemic stroke, a leading cause of long-term disability and care dependency in Japan.^[14]^ Approximately 47% of these patients are discharged home after an average acute hospital stay of 15 days,^[15]^ yet many continue to experience long-term physical or cognitive impairment.^[16,17]^ Transitional care is particularly important for this population due to their high risk of readmission from recurrent stroke or related complications.^[18,19]^ Although previous research has demonstrated the benefits of individual transitional care interventions, such as improved quality of life, functional recovery, and reduced caregiver burden,^[20,21]^ evidence on how multiple services are combined for patients with ischemic stroke in routine practice remains limited.

Furthermore, acute stroke care implementation substantially varies across hospitals and regions, depending on factors such as stroke unit availability, the presence of trained personnel, and institutional capacity to provide comprehensive stroke management.^[22-26]^ Despite growing policy and clinical interest in transitional care, the ways in which transitional care services are operationalized across Japanese acute care hospitals – specifically whether they form coherent combinations of services or vary according to hospital-level characteristics – have not been systematically described.

Therefore, we conducted a descriptive, exploratory study to identify distinct patterns of transitional care service implementation among patients with ischemic stroke and to assess how these patterns are associated with hospital-level structural characteristics.

## 2. Materials and methods

### 2.1. Study design

This retrospective observational study used a nationwide hospital administrative claims database from Japan Medical Data Center (JMDC) Inc. The observation period was between April 2022 and August 2023. This manuscript adheres to the Strengthening the Reporting of Observational studies in Epidemiology and Reporting of studies Conducted using Observational Routinely collected Data guidelines.

### 2.2. Ethical considerations

This study was approved by the Ethics Committee of the National Center for Geriatrics and Gerontology (approval number: 1752). Informed consent was waived due to the use of anonymized data without personal information. In addition, patients were not directly involved.

### 2.3. Data aource

Data were obtained from the JMDC hospital-based claims database. As of 2023, the database includes claims and diagnosis procedure combination (DPC) data from 670 hospitals – approximately 8.0% of all Japanese hospitals – and contains about 22 million inpatient and outpatient records. Data are collected irrespective of patients’ insurance type and are anonymized and standardized using uniform coding systems.^[27]^

### 2.4. Participants

We included patients aged ≥40 years diagnosed with ischemic stroke (International Classification of Diseases, 10th Revision code: I63) who were admitted to, and discharged from, acute care hospitals in the DPC system, either to their homes or long-term care facilities. Those with hospital stays ≤3 days were excluded since they were considered unlikely to require transitional care.^[28]^

The DPC is a Japanese case-mix classification and partial bundled payment system for inpatient reimbursement. It categorizes hospitalizations by primary diagnoses and medical procedures, combining per-diem bundled payments with fee-for-service components.^[29]^ As of 2022, >80% of acute care hospital beds in Japan were covered under the DPC system.

### 2.5. Measurements

Transitional care services are included in the national tariff schedule under the Japanese public healthcare insurance system and are therefore identifiable as reimbursable medical services in administrative claims data. We initially identified all transitional care-related services eligible for reimbursement and then assessed whether claims were submitted for each service within the study population during the observation period. Only services for which at least 1 claim was observed were included in the final analysis, while services without any observed claims were excluded.

Based on this process, we identified 10 relevant transitional care services. By team consensus, these services were grouped into the following 4 categories: discharge planning (codes: 190192310 and 190192510), information transfer to post-discharge providers (codes: 180016110, 113030170, and 113032470), collaboration and coordination with post-discharge providers (codes: 113008910, 113010670, and 113011710), and patient and caregiver education (codes: 190056910, 113017610, 113017710, 113017810, 113017910, and 113701910).

We evaluated individual- and hospital-level characteristics to examine associations with transitional care service implementation patterns. Individual-level characteristics included age, sex, and Charlson comorbidity index (CCI) scores. CCI was calculated from all diagnoses within 1 year before index hospitalization using a validated Japanese algorithm.^[30]^ Hospital-level characteristics included the number of beds (20–99, 100–199, 200–299, 300–499, and ≥500 beds), hospital ownership type (national/public, university, and other), and nurse-to-patient ratio (7:1 and 10:1). All variables were treated as categorical.

### 2.6. Statistical analysis

Baseline characteristics (means, standard deviations, medians, interquartile ranges, frequencies, and proportions) were summarized using descriptive statistics. All statistical analyses were conducted using R version 4.3.1 (R Foundation for Statistical Computing Vienna, Austria), and 2-tailed *P*-values < 0.05 were considered statistically significant.

### 2.7. Identifying the patterns of transitional care service implementation

Latent class analysis (LCA) was used to identify unobserved patterns of transitional care. It probabilistically assigns individuals to classes based on categorical indicators.^[31]^ In this study, the indicators were the following 4 binary variables representing the presence or absence of each service category: discharge planning, information transfer, collaboration and coordination, and patient/caregiver education. Analysis was conducted with the poLCA package in R using the expectation–maximization algorithm for maximum likelihood estimation.^[32]^ Models were estimated 100 times with random starting values (nrep = 100), allowing up to 5000 iterations (maxiter = 5000). The solution with the highest log-likelihood was retained.

Models with 2 to 5 classes were compared based on model fit and interpretability. Fit was evaluated with the Akaike information criterion and Bayesian information criterion. The final model was selected based on fit statistics and interpretability. Patients were assigned to the class with the highest posterior probability.

### 2.8. Characterizing the patterns of transitional care service implementation

A conditional inference tree was used to examine the associations between LCA-defined patterns and individual- and hospital-level factors. This nonparametric method, implemented through the ctree2 function in the caret package,^[33,34]^ applies conditional inference theory to reduce variable selection bias and overfitting. Explanatory variables included patient age, sex, CCI score, hospital size, ownership type, and nurse-to-patient ratio. Model stability was assessed using 5-fold cross-validation.

### 2.9. Sensitivity analysis

We performed a sensitivity analysis using fractional logit regression to address uncertainty in class assignments. Posterior probabilities of class membership served as dependent variables instead of discrete class assignments, producing continuous outcomes bounded between 0 and 1. This approach has been validated in prior econometric literature.^[35]^ We regressed membership probabilities on explanatory variables to assess each factor’s contribution – particularly hospital-level characteristics – while accounting for classification uncertainty. This method helps reduce potential misclassification bias.^[30]^

## 3. Results

### 3.1. Participant characteristics

**Figure 1.**
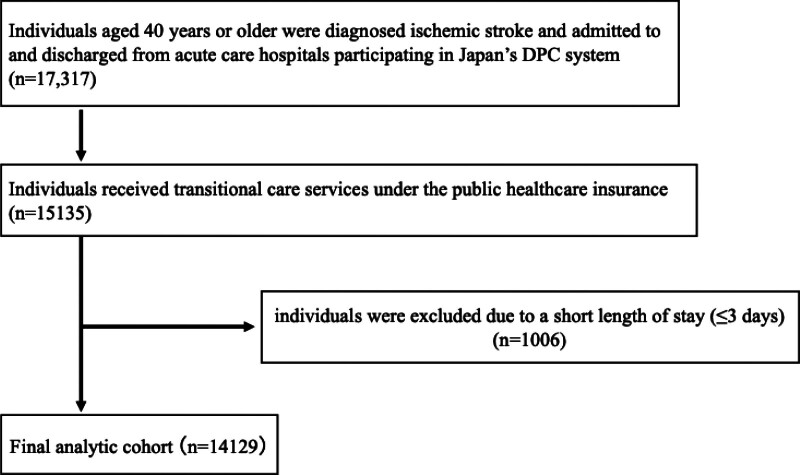
Participant flowchart. Flow diagram of participant selection between April 1, 2022 and August 31, 2023, showing the derivation of the final analytic cohort of 14,129 individuals with ischemic stroke admitted to and discharged from hospitals. DPC = diagnosis procedure combination.

Between April 1, 2022 and August 31, 2023, a total of 17,317 individuals aged ≥40 years diagnosed with ischemic stroke were admitted to and discharged from acute care hospitals participating in Japan’s DPC system. Among these, 15,135 received transitional care services under public healthcare insurance. A total of 1006 individuals were excluded because their hospital stay was ≤3 days. Ultimately, the final analytic cohort comprised 14,129 individuals (Fig. 1). Table 1 shows the participant characteristics.

**Table 1:** Participant characteristics.

Variable	Value
Age, median (IQR) years	77.0 (15.0)
Sex, n (%)	
Male	8480 (60.0)
Female	5649 (40.0)
Charlson comorbidity index scores, median (IQR)	2.0 (4.0)
Number of beds, n (%)	
20–99	130 (0.9)
100–199	1497 (11.0)
300–499	2568 (18.1)
≥500	6410 (45.3)
Hospital ownership type, n (%)	
National/public hospitals	1782 (12.6)
University hospitals	147 (1.0)
Other hospitals	12,200 (86.3)
Nurse-to-patient ratio, n (%)	
7:1	12,750 (90.2)
10:1	1379 (10.0)

IQR = interquartile range.

### 3.2. Identifying the patterns of transitional care service implementation

**Figure 2.**
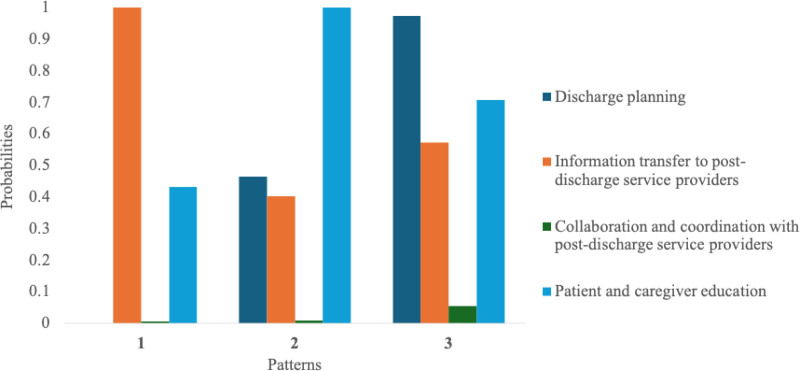
Three distinct patterns of transitional care service implementation identified by LCA. LCA = latent class analysis.

LCA identified 3 distinct transitional care service implementation patterns (Fig. 2 and Table 2), with model selection supported by Akaike information criterion and Bayesian information criterion values (Supplemental Digital Content 1, Supplemental Digital Content, https://links.lww.com/MD/R573). Pattern 1 was characterized by a high prevalence of information transfer, primarily involving the unidirectional sharing of patients’ clinical status, nutritional condition, dietary intake, and medication profiles with post-discharge healthcare and long-term care providers.

**Table 2:** Estimated probability of transitional care service implementation by pattern (services characterizing each pattern).

Estimated probability	Pattern 1	Pattern 2	Pattern 3
Discharge planning	0.00	0.46	0.97
Information transfer to post-discharge service providers	1.00	0.40	0.57
Collaboration and coordination with post-discharge service providers	0.00	0.01	0.05
Patient and caregiver education	0.43	1.00	0.71

Values represent estimated probabilities from latent class analysis and do not indicate deterministic implementation. Each value reflects the probability of service implementation within each pattern.

Pattern 2 emphasized patient and caregiver education. This included guidance on maintaining and improving physical function and activities of daily living, instruction in caregiving techniques, home modification recommendations, and explanations of medications prescribed during hospitalization, with attention to potential side effects, interactions, and risks of duplication. Nutritional counseling during hospitalization was also frequently included.

Pattern 3 was defined by the frequent provision of discharge planning services, along with a higher collaborative coordination rate with post-discharge providers than the other patterns. Discharge planning involved the early identification of patients with complex care needs and the development of individualized discharge plans, typically initiated within 7 days of admission, in accordance with national reimbursement requirements. Collaborative coordination entailed structured, multidisciplinary collaboration between hospital staff and community-based healthcare or long-term care providers to jointly deliver discharge instructions and share essential information, such as care plans, medication details, and dietary guidance, to ensure continuity of care.

### 3.3. Characterizing the patterns of transitional care service implementation

**Figure 3.**
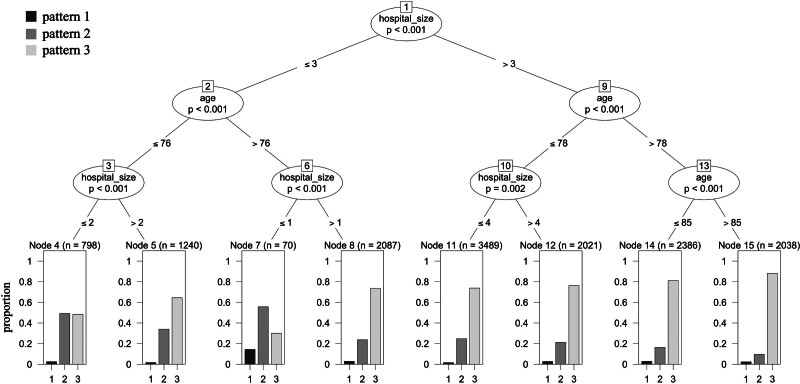
Conditional inference tree analysis results. Conditional inference tree analysis results showing the relationships of hospital size and patient age with the 3 patterns of transitional care service implementation.

Figure 3 shows the conditional inference tree analysis results. Patients admitted to larger hospitals and those of older age were more likely to fall into pattern 3. Those admitted to smaller hospitals were more frequently classified into pattern 1 or 2.

### 3.4. Sensitivity analysis

Fractional logit regression results are shown in the Tables, Supplemental Digital Contents 2 to 4, Supplemental Digital Content, https://links.lww.com/MD/R573. These findings aligned with the conditional inference tree analysis, showing the number of beds as a significant predictor of pattern membership.

## 4. Discussion

We identified 3 distinct patterns of transitional care service implementation across Japanese acute care hospitals using latent class analysis and a conditional inference tree. These patterns differed primarily in the composition and intensity of services delivered, as well as in their distribution across hospital types.

Hospitals classified as pattern 3 were characterized by a higher likelihood of providing a broader set of transitional care services, including discharge planning and structured coordination with post-discharge providers, and were more frequently large institutions. In contrast, hospitals in patterns 1 and 2 tended to focus on patient education and information transfer activities, which generally require fewer institutional resources and were more commonly observed among smaller hospitals.

Studies from the United States have shown that transitional care service implementation varies with hospital characteristics, including size, ownership, geographic location, and participation in value-based payment models such as Accountable Care Organizations.^[8,36,37]^ Although Japan operates under a universal health insurance system without market-based payment differentiation or value-based incentives, our findings are conceptually consistent with these studies in demonstrating that transitional care service implementation varies systematically according to hospital-level structural characteristics.

The patterns identified in this study appear to reflect institutional and workforce constraints that shape how transitional care services are operationalized in routine practice. In Japan, discharge planning is a reimbursable service that requires early identification of patients with complex needs and the establishment of a dedicated department staffed by care coordinators. Structured coordination with post-discharge providers is reimbursed separately and entails systematic information exchange and multidisciplinary collaboration. These requirements place significant demands on staffing, time, and inter-organizational infrastructure. Smaller hospitals with limited resources may find compliance challenging, prompting the Ministry of Health, Labour, and Welfare in 2022 to revise eligibility standards for discharge planning in underserved areas.^[38]^ Furthermore, government reports indicate that approximately 40% of acute care hospitals lack sufficient staff to fully implement transitional care services,^[39]^ and the availability of dedicated discharge coordinators declines markedly with hospital size.^[40]^

In Japan, the quality of acute stroke care – such as tissue plasminogen activator administration and stroke care units availability – has been shown to correlate with hospital characteristics, including bed capacity, staffing levels, and patient volume.^[25,26,41]^ In a similar vein, the patterns identified in this study should be interpreted as reflecting differences in organizational and structural capacity for implementing transitional care services, rather than differences in the quality or effectiveness of care. These results underscore the importance of considering hospital resources when developing strategies to improve the continuity of stroke care.

Whether these patterns are optimal remains uncertain. Prior evidence suggests that combinations of transitional care strategies may be associated with patient outcomes. For example, Figueroa et al reported a positive association between the number of transitional care strategies implemented and patient satisfaction,^[42]^ and Mays et al found that hospitals engaging in cross-setting information exchange, when combined with at least 2 additional strategies, achieved greater reductions in readmission rates.^[10]^ Comparable evaluations remain limited in Japan. Our findings may serve as a basis for future research to inform evaluations of how different patterns of transitional care service implementation relate to patient outcomes.

This study has several limitations. First, the dataset did not include all acute care hospitals in Japan, regardless of DPC participation; therefore, the findings may not be fully generalizable to all patients hospitalized with ischemic stroke.

Second, the study did not include patient-level outcome data, such as readmissions, functional status, or mortality. As a result, the identified transitional care service implementation patterns should not be interpreted as reflecting differences in effectiveness, quality, or clinical benefit. In particular, the absence of outcome data precludes any conclusions regarding the effectiveness or superiority of pattern 3 or other patterns.

Third, the identification of transitional care services was based on claims data, which may not fully capture services delivered but not billed. Some hospitals may have provided transitional care services without submitting claims, potentially leading to an underestimation of implementation.

Fourth, several potentially important hospital-level characteristics were unavailable, including geographic location, financial status, relationships with community-based providers, participation in networks, and staffing levels. In particular, geographic location may be relevant, as regional disparities in healthcare workforce distribution may have influenced the observed implementation patterns by shaping hospitals’ organizational and human resource capacity to deliver transitional care services. Physicians and nurses are disproportionately concentrated in large urban hospitals, while rural facilities continue to face chronic shortages.^[43,44]^ Because detailed geographic identifiers (e.g., urban versus rural location or prefecture-level information) were unavailable in the present dataset, it was not possible to distinguish whether the observed variation in transitional care service implementation reflects hospital-level organizational characteristics, regional healthcare constraints, or a combination of both. Accordingly, further investigation using data sources that incorporate geographic information will be necessary to more precisely evaluate the role of regional context in shaping transitional care service implementation in Japan.

Fifth, the latent patterns of transitional care service identified represent statistical groupings based on model-derived probabilities, rather than mutually exclusive categories. To address potential misclassification, we performed sensitivity analyses using membership probabilities in regression models, which supported our main findings’ robustness.

Despite these limitations, this study provides novel insights into transitional care service implementation across Japanese acute care hospitals and highlights structural variation in service provision. Rather than indicating differential effectiveness, the findings underscore heterogeneity in implementation capacity and access to transitional care services across hospitals.

In conclusion, this study identified 3 distinct transitional care service implementation patterns in Japanese acute care hospitals, associated with hospital-level characteristics, particularly the number of beds. Although causal inferences cannot be drawn, these findings suggest that organizational and resource-related factors contribute to heterogeneity in transitional care service delivery.

As Japan faces rapid population aging and widening regional disparities in healthcare infrastructure, addressing barriers to comprehensive transitional care – particularly in smaller or resource-constrained hospitals – remains a critical priority for policy and practice. Future research should examine how different combinations of transitional care service strategies influence patient outcomes and identify models that foster more equitable and effective hospital–home transitions.

## Acknowledgments

Generative artificial intelligence was used to refine English sentences when translating from Japanese to English. The final version was reviewed by a professional English-language editing company. We thank Editage (www.editage.jp) for English-language editing.

## Author contributions

**Conceptualization:** Naoki Takashi, Misaki Fujisawa, Tomoko Ohura, Joji Onishi, Shosuke Ohtera.

**Data curation:** Naoki Takashi.

**Formal analysis:** Naoki Takashi.

**Funding acquisition:** Naoki Takashi, Shosuke Ohtera.

**Investigation:** Naoki Takashi.

**Methodology:** Naoki Takashi, Misaki Fujisawa, Tomoko Ohura, Shosuke Ohtera.

**Project administration:** Naoki Takashi.

**Supervision:** Joji Onishi, Shosuke Ohtera.

**Writing – original draft:** Naoki Takashi.

**Writing – review & editing:** Misaki Fujisawa, Tomoko Ohura, Joji Onishi, Shosuke Ohtera.

## Supplementary Material


