# Supplementary material for: Variation in transitional care implementation patterns for older adults with stroke in Japan: A retrospective observational study
Source: Medicine (Baltimore). 2026 Mar 27;105(13):e48161. doi: 10.1097/MD.0000000000048161 (PMC13034975; doi:10.1097/MD.0000000000048161)
Supplement: Supplementary file 1 [file medi-105-e48161-s001.docx]

Supplementary Material

**Supplemental Digital Content 1. AIC and BIC values for the 2–5 class latent class models**

| Class model | AIC | BIC | Notes |
| --- | --- | --- | --- |
| 2 class | 53173.62 | 53241.62 |  |
| 3 class | 52961.37 | 53067.15 | Selected model |
| 4 class | NA | NA | Model not estimable (negative degrees of freedom) |
| 5 class | NA | NA | Model not estimable (negative degrees of freedom) |
| Models with 4 and 5 classes resulted in negative degrees of freedom due to model overparameterization and were therefore not estimable.  **Supplemental Digital Content 2. Results of fractional logit regression for the posterior probability of Pattern 1**   \| R-squared (pseudo): 0.003 \| \| \| \| \| \| \| \| --- \| --- \| --- \| --- \| --- \| --- \| --- \| \| Variable \| Estimate \| \| Standard Error \| \| p-value \| \| \| (Intercept) \| -2.78 \|  \| 0.23 \|  \| <0.001 \|  \| \| Number of beds \| -0.12 \|  \| 0.042 \|  \| 0.005 \|  \| \| Hospital ownership type \| -0.08 \|  \| 0.050 \|  \| 0.135 \|  \| \| Nurse-to-patient ratio \| 0.21 \|  \| 0.110 \|  \| 0.060 \|  \| \| Age \| 0.06 \|  \| 0.034 \|  \| 0.107 \|  \| \| Sex \| 0.17 \|  \| 0.071 \|  \| 0.015 \|  \| \| Charlson Comorbidity Index scores \| -0.06 \|  \| 0.037 \|  \| 0.108 \|  \|   Supplemental Digital Content 2. Results of fractional logit regression for the posterior probability of Pattern 1. Estimated associations between hospital- and patient-level characteristics and the probability of belonging to Pattern 1.  **Supplemental Digital Content 3. Results of fractional logit regression for the posterior probability of Pattern 2**   \| R-squared (pseudo): 0.082 \| \| \| \| \| \| \| \| --- \| --- \| --- \| --- \| --- \| --- \| --- \| \| Variable \| Estimate \| \| Standard Error \| \| p-value \| \| \| (Intercept) \| 0.36 \|  \| 0.074 \|  \| <0.001 \|  \| \| Number of beds \| -0.25 \|  \| 0.013 \|  \| <0.001 \|  \| \| Hospital ownership type \| -0.01 \|  \| 0.017 \|  \| 0.692 \|  \| \| Nurse-to-patient ratio \| 0.03 \|  \| 0.041 \|  \| 0.496 \|  \| \| Age \| -0.30 \|  \| 0.011 \|  \| <0.001 \|  \| \| Sex \| 0.12 \|  \| 0.023 \|  \| <0.001 \|  \| \| Charlson Comorbidity Index scores \| 0.03 \|  \| 0.011 \|  \| 0.003 \|  \|   Supplemental Digital Content 3. Results of fractional logit regression for the posterior probability of Pattern 2. Estimated associations between hospital- and patient-level characteristics and the probability of belonging to Pattern 2.  **Supplemental Digital Content 4. Results of fractional logit regression for the posterior probability of Pattern 3**   \| R-squared (pseudo): 0.074 \| \| \| \| \| \| \| \| --- \| --- \| --- \| --- \| --- \| --- \| --- \| \| Variable \| Estimate \| \| Standard Error \| \| p-value \| \| \| (Intercept) \| -0.57 \|  \| 0.078 \|  \| <0.001 \|  \| \| Number of beds \| 0.26 \|  \| 0.014 \|  \| <0.001 \|  \| \| Hospital ownership type \| 0.02 \|  \| 0.018 \|  \| 0.326 \|  \| \| Nurse-to-patient ratio \| -0.06 \|  \| 0.043 \|  \| 0.141 \|  \| \| Age \| 0.28 \|  \| 0.012 \|  \| <0.001 \|  \| \| Sex \| -0.14 \|  \| 0.025 \|  \| <0.001 \|  \| \| Charlson Comorbidity Index scores \| -0.02 \|  \| 0.012 \|  \| 0.041 \|  \|   Supplemental Digital Content 4. Results of fractional logit regression for the posterior probability of Pattern 3. Estimated associations between hospital- and patient-level characteristics and the probability of belonging to Pattern 3. | | | |
